# Co-infections of rickettsiales in clinically healthy, *Leishmania infantum* seropositive and seronegative dogs: a systematic literature review and new findings from Southern Italy

**DOI:** 10.1007/s00436-025-08458-4

**Published:** 2025-01-29

**Authors:** Oana Gusatoaia, Livia Perles, Maria Alfonsa Cavalera, Annamaria Uva, Floriana Gernone, Domenico Otranto, Andrea Zatelli

**Affiliations:** 1 Department of Veterinary Medicine, University of Bari, Valenzano, Italy; 2 Department of Veterinary Clinical Sciences, City University of Hong Kong, Hong Kong SAR, China

**Keywords:** *Ehrlichia canis*, *Anaplasma phagocytophilum*, *Leishmania infantum*, Canine vector-borne disease, Co-infection, Serology

## Abstract

Canine leishmaniosis (CanL), caused by *Leishmania infantum*, is a widespread vector-borne disease. In Italy, an endemic region for CanL, overlapping transmission of *L. infantum* and tick-borne pathogens (TBPs) like *Anaplasma phagocytophilum* and *Ehrlichia canis* is increasingly reported. Dogs with clinical leishmaniosis often show higher co-infection rates and pronounced clinicopathological abnormalities. This study presents a systematic literature review and new findings from southern Italy, focusing on co-infections with *E. canis* and *A. phagocytophilum* in clinically healthy *L. infantum* seropositive and seronegative dogs. The systematic review identified two eligible studies. The first reported 34/488 (7%) dogs *L. infantum* seropositive, with 11.8% also seropositive for *A. phagocytophilum*. Among 454 seronegative dogs, 3% were seropositive for *A. phagocytophilum* and 2.4% for *E. canis*. The second study identified 154/1260 (12.2%) dogs *L. infantum* seropositive, with co-infection rates of 0.6% and 1.9% for *A. phagocytophilum* and *E. canis*, respectively. Among 1106 seronegative dogs, 1.3% were seropositive for *A. phagocytophilum* and 2.3% for *E. canis*. In the retrospective study from southern Italy, 90/154 (58.4%) dogs were *L. infantum* seropositive, with co-infection rates of 4.4% for *A. phagocytophilum* and 2.2% for *A. phagocytophilum* and *E. canis*. Among 64 seronegative dogs, 1.6% showed similar co-infections. This is the first systematic review in Italy, documenting low and comparable co-infection rates with *A. phagocytophilum* and *E. canis* in clinically healthy dogs, regardless of *L. infantum* serostatus. These findings suggest that co-infections may occur independently, offering insights into vector-borne disease dynamics in endemic areas.

## Introduction

Canine vector-borne diseases (CVBDs) are a group of infectious diseases caused by protozoa, helminths, bacteria, and viruses and transmitted by blood-feeding arthropods including ticks, fleas, lice, and phlebotomine sand flies. To date, CVBDs are among the most important health issues affecting dogs worldwide (Otranto and Dantas-Torres 2010). Within the CVBDs, canine leishmaniosis (CanL) by *Leishmania infantum* is considered one of the most important diseases of zoonotic concern in Europe, being endemic in the Mediterranean regions with prevalence rates of infection up to 60% in exposed populations (Baneth et al. 2008; Zini et al. 2020; European Scientific Counsel Companion Animal Parasites (ESCCAP) 2023). Furthermore, an increasing trend in the number of dogs with no travel history in CanL endemic countries has been reported in central and northern Europe (Maia and Cardoso 2015), previously considered to be CanL-free such as Netherlands (Slappendel 1988; Dìaz-Espineira and Slappendel 1997), Germany (Naucke and Lorentz 2012), United Kingdom (Shaw et al. 2009), Hungary (Tánczos et al. 2012), Romania (Mircean et al. 2014), Finland (Karkamo et al. 2014). In this context, Italy belongs to the historically CanL endemic countries with an average percentage of *L. infantum* seroprevalence of 18.65% in 2017 (Italian Ministry of Health 2021) up to 28.2% in southern Italy and islands, 29.6% in central Italy and 21.6% in north Italy (Mendoza-Roldan et al. 2020).

However, in Italy, as well as in many other endemic areas for CVBDs across the World, the presence of *L. infantum* and tick-borne pathogens (TBPs) may overlap due to similar transmission periods of their vectors (Mekuzas et al. 2009). For instance, ticks in Italy transmit various vector-borne pathogens (VBPs), including *Anaplasma phagocytophilum*, a Gram-negative coccus, *Anaplasma platys*, a Gram negative bacterium and *Ehrlichia canis,* an obligate intracellular bacterium (Ebani et al. 2014) with prevalence of *A. phagocytophilum* and *E. canis* up to 3.3% and ranging from 0.9 to 3.3%, respectively (De la Fuente et al. 2006; Vascellari et al. 2016).

From a clinical perspective, activation of the host immune mechanisms by *L. infantum* and *E. canis* can induce a variety of immunopathological responses, characterized by frequently overlapping clinical signs (e.g., lymph node enlargement, weight loss, and splenomegaly) (De Tommasi et al. 2013) and laboratory abnormalities such as anemia, thrombocytopenia, hyperglobulinemia, hypoalbuminemia, mild increase in hepatic enzymes (De Tommasi et al. 2013; Sainz et al. 2015; Solano-Gallego et al. 2015; Paltrinieri et al. 2016). In addition, it is suggested that dogs with clinical leishmaniosis have a higher rate of co-infections with other TBPs such *E. canis*, *A. phagocytophilum* and/or *A. platys* when compared to healthy dogs, and more pronounced clinicopathological abnormalities (Baxarias et al. 2018).

Currently, there is no available data on the potentially higher rate of co-infections with other VBPs such as *Ehrlichia* spp*.* and *Anaplasma* spp*.* in clinically healthy *L. infantum* seropositive dogs. For unique geographical, climate and habitat diversity features, Italy is a hotspot for CanL and for thriving arthropod vectors, along with their associated pathogens (Otranto and Dantas-Torres 2010; Genovesi et al. 2014). Therefore, in order to obtain a better understanding of the relationship between *L. infantum* seropositivity and co-infections with other VBPs we provide a systematic literature review on the occurrence of co-infections with *E. canis, A. phagocytophilum* in clinically healthy *L. infantum* seropositive and seronegative dogs from Italy, along with data from a cohort of dogs in southern Italy.

## Methods

### Systematic literature review

#### Search strategy

On 19th of September 2023, a systematic literature search was conducted according to the Preferred Reporting Items for Systematic reviews and Meta-Analyses (PRISMA) 2020 statement (Page et al. 2021). Four online literature databases (i.e., BASE, Google Scholar, PubMed and WoS) were screened to identify all publications on the prevalence of co-infections with *Ehrlichia* spp*.*, *Anaplasma* spp*.* and *Leishmania* spp. in Italy. The keywords “anaplasma,” “canine,” “dog,” “ehrlichia,” “epidemiological,” “epidemiology,” “Italy,” “leishmania,” “leishmaniasis,” “leishmaniosis,” and “prevalence” were used. The literature search on BASE, Google Scholar, and PubMed was split into two searches and was performed using Boolean Operators AND and OR as follows: [(prevalence OR epidemiology OR epidemiological) AND (canine OR dog) AND (anaplasma AND/OR ehrlichia) AND (Italy)] followed by [(prevalence OR epidemiology OR epidemiological) AND (canine OR dog) AND (anaplasma AND/OR ehrlichia) AND (Italy) AND (leishmania OR leishmaniosis OR leishmaniasis)]. On WoS database, since the forward slash sign (“/”) is not accepted, four different researches were made, using the Boolean Operators AND and OR as follows: [(prevalence OR epidemiology OR epidemiological) AND (canine OR dog) AND (anaplasma AND ehrlichia) AND (Italy)], [(prevalence OR epidemiology OR epidemiological) AND (canine OR dog) AND (anaplasma AND ehrlichia) AND (Italy) AND (leishmania OR leishmaniosis OR leishmaniasis)], [(prevalence OR epidemiology OR epidemiological) AND (canine OR dog) AND (anaplasma OR ehrlichia) AND (Italy)] and [(prevalence OR epidemiology OR epidemiological) AND (canine OR dog) AND (anaplasma OR ehrlichia) AND (Italy) AND (leishmania OR leishmaniosis OR leishmaniasis)].

### Inclusion and exclusion criteria

To be considered, the articles were required to meet the following inclusion criteria: (i) should involve studies conducted in dogs from Italy; (ii) should involve clinically healthy dogs; (iii) should include the use of serological tests to detect the presence of antibodies anti-*Leishmania* spp. (i.e., immunofluorescence antibody test [IFAT] and enzyme-linked immunosorbent assay [ELISA]), anti-*Ehrlichia* spp. and anti-*Anaplasma* spp. (IFAT); (iv) should be available into databases between the 1st of January 2000 to 19th of September 2023; (v) should have accessible abstract; (vi) should be available in Italian, English, French, Spanish, and Portuguese languages. Any type of original article, PhD thesis and conference proceedings were also considered eligible.

Studies were excluded if incomplete data to evaluate the occurrence of *Anaplasma* spp*.* and/or *Ehrlichia* spp*.* in *L. infantum* seropositive dogs was present, if screenings were not carried out in Italy, and the presence of clinical signs compatible with leishmaniosis, anaplasmosis, and ehrlichiosis were reported in the enrolled dogs.

### Selection process

The entire selection process was made in a blinded manner by two independent reviewers (OG, LP). After removing duplicates found in databases, the titles and abstracts of each document were reviewed based on the inclusion and exclusion criteria. The selected publications were read in full by both reviewers to either confirm their eligibility and extract the data or to exclude them. When the selection process was concluded, if present, any disagreement was discussed and resolved by consensus.

### Data extraction

Using a Microsoft Excel® spreadsheet, the following data was extracted and recorded from the included studies: title, first author name, year of publication, journal, type of article, type of study, Italian area where studies were conducted, sample size, dog signalment, diagnostic methods used for screening, number of positive dogs for *Anaplasma* spp., *Ehrlichia* spp., and *Leishmania* spp., number of positive dogs co-infected with two or all three aforementioned VBPs.

### Study design and availability of data

Medical records of dogs, of any age, sex, and breed which were referred to the Medical Clinic Unit of the Department of Veterinary Medicine (Valenzano, Italy) and clinically evaluated in recently published clinico-parasitological trials (Cavalera et al. 2022) or still ongoing (data unpublished) were retrospectively collected. Dogs were considered eligible for inclusion in this study if they were clinically healthy (i.e., physical examination and laboratory findings [complete blood count, serum biochemical analysis, and serum electrophoresis] unremarkable), tested for *L. infantum*, *E. canis*, and *Anaplasma* spp. by indirect immunofluorescent antibody test (IFAT), residing in Italy and if they had available data on treatments received.

Dogs were excluded if they showed clinical signs compatible with leishmaniosis, ehrlichiosis or anaplasmosis (e.g., thinning, dermatitis with alopecia or dandruff, onychogryphosis, epistaxis, uveitis, and reactive or enlarged explorable lymph nodes) (De Tommasi et al. 2013) and IFAT test results for *L. infantum*, *E. canis*, and *Anaplasma* spp. were not available.

To compare the proportions of animals seropositive for *A. phagocytophilum* and/or *E. canis* between dogs seropositive and seronegative for *L. infantum*, a chi-square test was employed. A value of *P* < 0.05 was considered statistically significant. The statistical analyses were performed using R Core Team, version 4.3.3 (R Foundation for Statistical Computing, Vienna, Austria).

## Results

### Literature search results

**Fig. 1:**
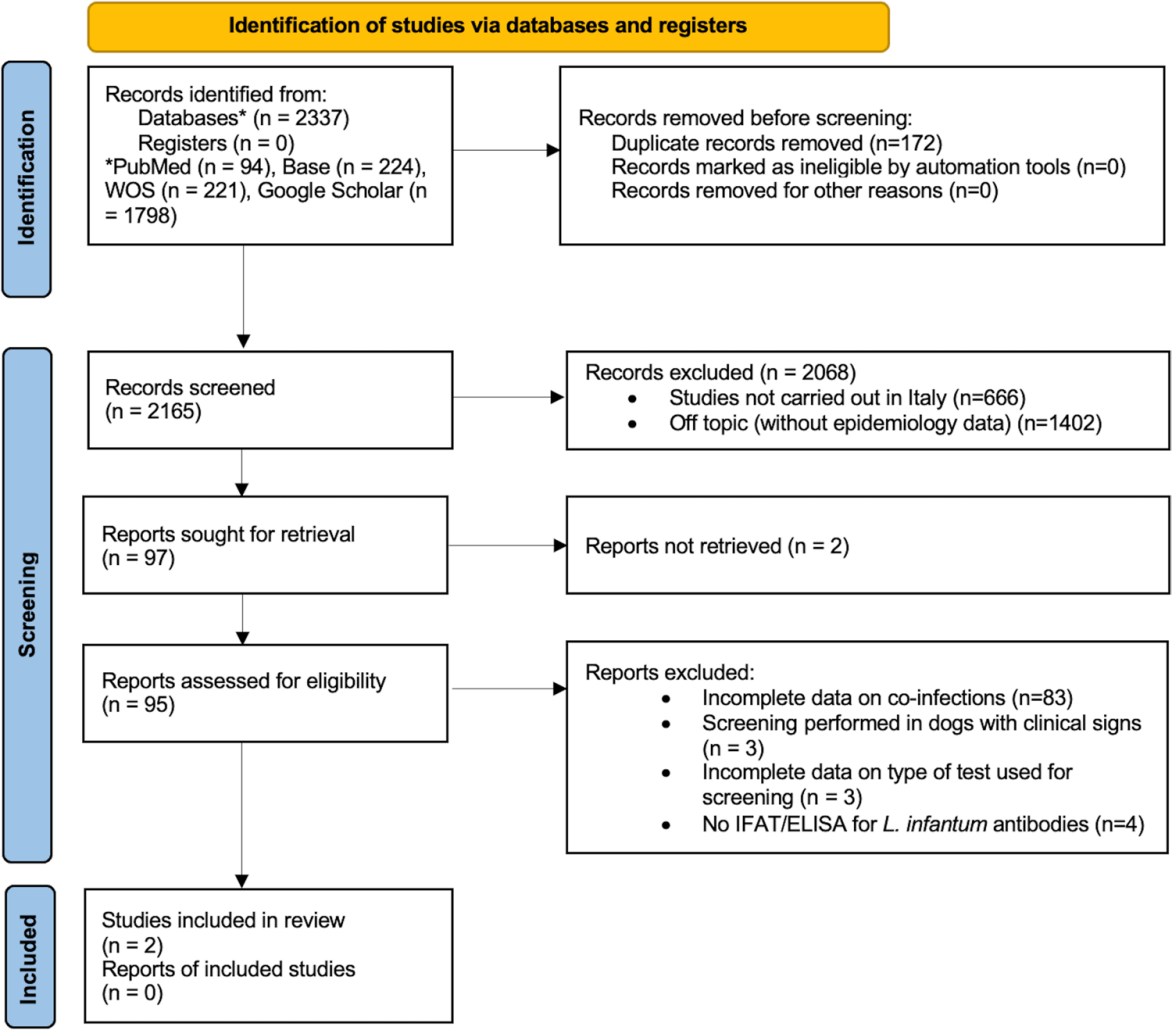
PRISMA 2020 flowchart illustrating the study selection process

The workflow of the literature research based on PRISMA guidelines for systematic reviews is shown in Fig. 1. The total number of records identified from databases was 2337. One hundred seventy-two articles were excluded because of duplication. After title/abstract screening, 2068 articles were excluded for the following reasons: studies conducted in other countries than Italy (*n* = 666) and studies without epidemiological data (*n* = 1402). The 97 references obtained were sought for retrieval and 2 reports were not retrieved. Ninety-five references were obtained and underwent full-text eligibility assessment. Out of these, 93 articles were excluded due to: incomplete data available on co-infections (*n* = 83), screenings performed in dogs with clinical signs (*n* = 3), incomplete data on diagnostic methods (*n* = 3), lack of serological test for *L. infantum* (*n* = 3), and use of in-clinic diagnostic kits (rapid tests) for screening (*n* = 1). Finally, 2 studies were found eligible and included in this systematic review.

The qualitative synthesis (first author, year, and type of publication, study type, number and type of enrolled dogs, study location, cut-off of the serological test used) of the two studies resulting from the systematic literature search is shown in Table 1.

**Table 1:** A qualitative synthesis of the two studies resulted eligible from the systematic literature search on co-infections by *Leishmania infantum* (*L. i.*), *Ehrlichia canis* (*E.c.*) and *Anaplasma phagocytophilum* (*A. p.)* in clinically healthy dogs in Italy

Author, year	Type of publication	Type of study	N of dogs enrolled	Part of Italy	Location (region)	Type (n) of dog population	Type of serologic test	Cut off *L. i*	Cut off *E. c*	Cut off *A. p*
Vascellari et al. 2016	Original article	Prospective	488	North, Northeast	Veneto, Lombardy, Emilia-Romagna	CBD	IFAT	1:40	1:50	1:80
						FRD				
Morganti et al. 2022	Original article	Retrospective, epidemiological longitudinal	1260	Central	Umbria	CBD	IFAT	1:80	1:25	1:80

*IFAT* indirect immuno-fluorescent antibody test; *N* number; *CBD* Candidate blood donors; *FRD* free roaming dogs

The serological positive results for *Ehrlichia* spp. and *Anaplasma* spp. were limited to *E. canis* and *A. phagocytophilum* respectively. The number of dogs that tested serologically positive and negative for *L. infantum* in each study, as well as the number of *E. canis* and/or *A. phagocytophilum* co-infected dogs according to serology for *L. infantum* is presented in Table 2.

**Table 2:** Number of dogs involved in the two studies resulting from the systematic literature review in addition to those enrolled in our study and for each of them number (percentage) of animals tested negative for all parasites considered in the studies, *Leishmania infantum* (*L. i.*) seronegative and seropositive**,** *Ehrlichia canis* (*E. c.*) and *Anaplasma phagocytophilum* (*A. p.)* seropositive as well as the number (percentage) of *E. canis* and/or *A. phagocytophilum* co-infected dogs according to serology for *L. infantum*

Author, year	Number of dogs enrolled	Negative for all tested pathogens (%)	*L. i.* seronegative (%)	*L. i.* seropositive (%)	*L. i.* seronegative dogs			*L. i.* seropositive dogs		
					*A. p*. seropos. (%)	*E. c.* seropos. (%)	*A. p.* and *E. c.* seropos. (%)	*A. p.* seropos. (%)	*E. c.* seropos. (%)	*A. p.* and *E. c.* seropos. (%)
Vascellari et al. 2016	488	429 (87.9)	454 (93.00)	34 (7.0)	14 (3.0)	11 (2.4)	-	4^1^ (11.8)	-	-
Morganti et al. 2022	1260	1066 (84.6)	1106 (87.8)	154 (12.2)	14 (1.3)	26 (2.3)	-	1 (0.6)	3 (1.9)	-
**Studied group**	154	56 (35.4)	64 (41.5)	90 (58.4)	1 (1.6)	-	1 (1.6)	4 (4.4)	-	2 (2.2)

^1^*L. infantum* antibody titer 1:40

In the publication by Vascellari et al. (2016), 34 (7.0%) out of 488 enrolled dogs were *L. infantum* seropositive. Four (11.8%) out of 34 dogs, were *A. phagocitophylum* seropositive. Among the *L. infantum* seronegative animals (*n* = 454; 93.0%), 14 (3.0%) were *A. phagocitophylum* seropositive and 11 (2.4%) were *E. canis* seropositive (Table 2).

In Morganti et al. (2022), 154 (12.2%) out of 1260 dogs enrolled were seropositive for *L. infantum*. Among them, one (0.6%) and 3 (1.9%) dogs were *A. phagocitophylum* and *E. canis* seropositive, respectively (Table 2). Out of 1260, 1106 (87.8%) were *L. infantum* seronegative dogs. Among them, 14 (1.3%) and 26 (2.3%) were *A. phagocytophilum* and *E. canis* seropositive, respectively.

*Leishmania infantum, A. phagocytophilum* and *E. canis* antibody titers according to the type of dog population (i.e., candidate blood donors, free roaming dogs) enrolled in the two articles resulting from the systematic literature review are described in Table 3.

**Table 3:** *Leishmania infantum* (*L. i.*),* Anaplasma phagocytophilum* (*A. p.*), and *Ehrlichia canis* (*E. c.*) antibody titers (Ab) according to the type of dog population enrolled in the two articles resulted from the systematic literature review

Author, year	Type of dog population	*L. i.* Ab titer (n)	*A. p.* Ab titer (n)	*E. c.* Ab titer (n)	*L. i.* and *A. p.* seropositive		*L. i.* and *E. c.* seropositive (n)		*A. p.* and *E. c.* seropositive (n)	
					*L. i.* Ab titer (n)	*A. p.* Ab titer (n)	*L. i.* Ab titer (n)	*E.c.* Ab titer (n)	*A. p.* Ab titer (n)	*E.c.* Ab titer (n)
Vascellari et al. 2016	FRD (n = 338)	1:40 (8)	1:80 (5)	1:50 (1)	1:40 (4)	1:80 (2) 1:160 (1) 1:320 (1)	-	-	-	-
		1:80 (1)	1:160 (2)	1:100 (1)						
		1:1280 (1)								
	CBD (n = 150)	1:40 (27)	1:80 (5)	1:50 (7)	-	-	-	-	-	-
			1:160 (3)	1:100 (1)						
			1:320 (2)	1:400 (1)						
			1:5120 (1)							
Morganti et al. 2022	CBD (n = 1260)	1:80 (95)	1:80 (7)	1:25 (17)	NA	NA	NA	NA	NA	NA
		1:160 (25)	1:160 (1)	1:50 (5)						
		1:320 (23)	1:320 (4)	1:100 (5)						
		1:640 (11)	1:640 (2)	1:200 (2)						

*NA* not available, *n* number, *CBD* candidate blood donors, *FRD* free roaming dogs

### Studied group

Records of 154 animals (i.e., *n* = 77 female and *n* = 77 male; 7.51 ± 3.65 years) met all the criteria and were enrolled in this study. Out of 154, 135 (87.7%) were shelter dogs, and 19 (12.3%) were hunting dogs. The number of dogs *L. infantum* seronegative and seropositive and the number of those co-infected with *E. canis* and/or *A*. *phagocytophilum* are presented in Table 2. Out of 154 dogs, 90 (58.4%) were found to be *L. infantum* seropositive by IFAT, of which 4 (4.4%) seropositive for *A. phagocytophilum*, and 2 (2.2%) for *E. canis* and *A. phagocytophilum.* None of the *L. infantum* seropositive dogs was *E. canis* seropositive only. Antibody titres for *L. infantum*, *A. phagocytophilum*, and *E. canis* in the 8 infected dogs are reported in Table 4.

**Table 4:** Antibody titres for *Leishmania infantum*, *Anaplasma phagocitophylum*, and *Ehrlichia canis* in the 8 infected dogs of the studied group

ID	*L. infantum* antibody titre	*A. phagocitophylum* antibody titre	*E. canis* antibody titre
#1	1:80	1:160	neg
#2	1:160	1:1280	1:2560
#3	1:640	1:80	neg
#4	1:640	1:1280	1:1280
#5	1:80	1:160	neg
#6	1:80	1:160	neg
#7	neg	1:80	neg
#8	neg	1:320	1:640

Out of the 154 dogs, 64 (41.6%) were *L. infantum* seronegative with one (1.6%) seropositive for *A. phagocytophilum*, and one (1.6%) for *E. canis* and *A. phagocytophilum* (Table 2)*.*

No statistically significant differences were found between the *L. infantum* seropositive and seronegative groups in the proportion of *A. phagocytophilum* seropositive (*X*^*2*^ = 0.9889, *p* = 0.32) and *E. canis* and *A. phagocytophilum* seropositive (*X*^*2*^ = 0.0852, *p* = 0.77) animals.

**Fig. 2:**
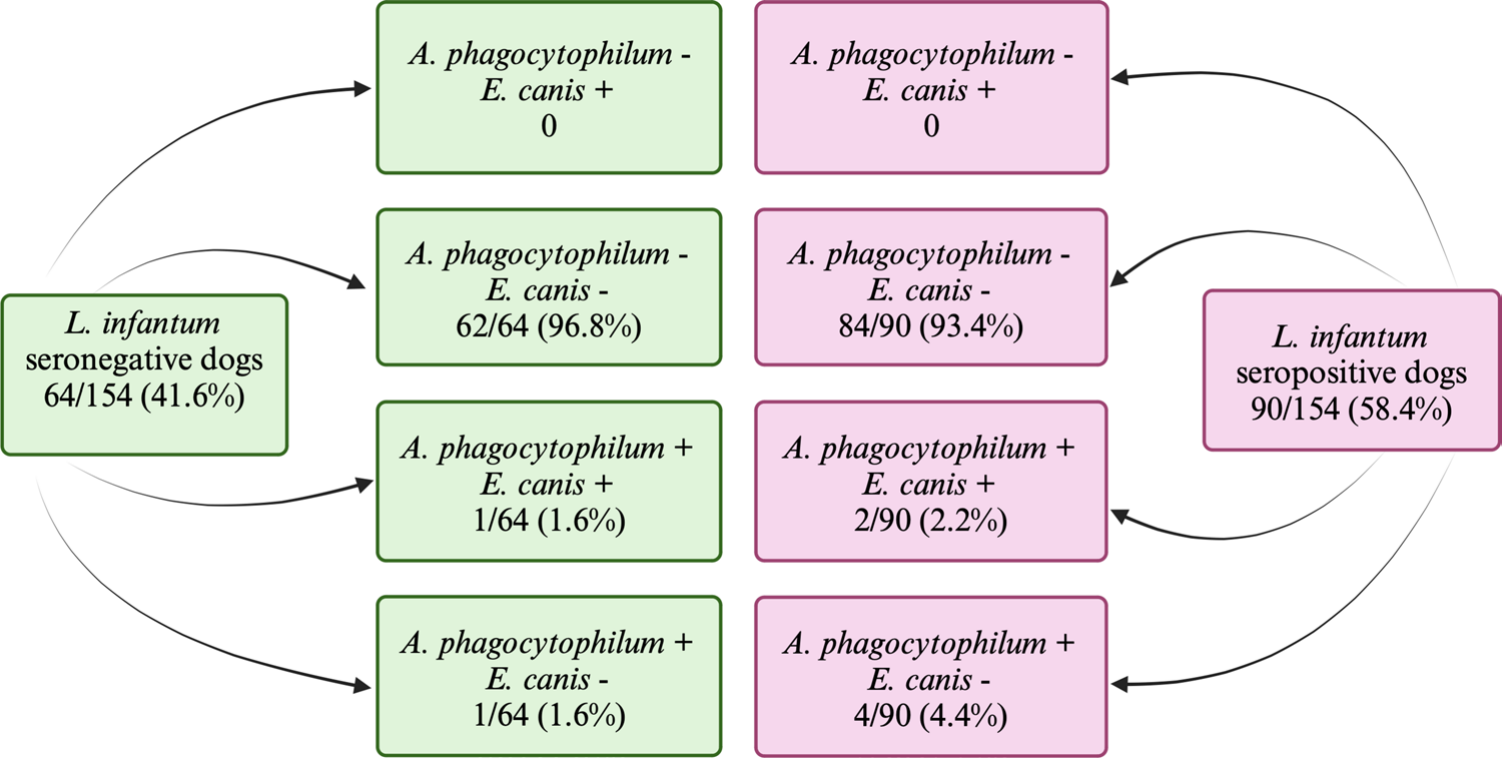
Positive (“ + “) and negative (“ – “) serologic results for *Ehrlichia* *canis* and/or *Anaplasma phagocytophilum* in *Leishmania infantum* serpopositive and seronegative dogs from the cohort studied

The serology results for *E. canis* and *A. phagocytophilum* in *L. infantum* seropositive and seronegative dogs expressed considering the entire population tested are reported in Fig. 2. No dogs were treated with anti-feeding/insecticidal products against the main vectors for ectoparasite control.

## Discussion

This study presents a systematic literature review on the occurrence of co-infections with *E. canis* and *A. phagocytophilum* in clinically healthy *L. infantum* seronegative and seropositive dogs from Italy. A total of two articles were identified as eligible for a qualitative synthesis. The studies by Vascellari et al. (2016) and Morganti et al. (2022) were considered. Furthermore, this article presents a retrospective study on the topic, providing data on dogs in the Apulian region.

Data from the systematic review suggest that limited information is available in the Italian peninsula due to the lack of research and inadequacy of data that did not meet the inclusion criteria. The first article considered for qualitative synthesis after the systematic literature review, by Vascellari et al. (2016), showed an overall prevalence of *L. infantum* of 7.0% with very similar prevalence between the two groups of dogs included in this study (i.e., FRD and CBD): 7.1% and 6.7% prevalence in FRD and CBD, respectively, as determined by IFAT*.* Co-infections with *L. infantum* and *Anaplasma* spp. was found in 4 (11.8%) dogs and none of dogs showed co-infection with *L. infantum–E. canis* or *L. infantum–E. canis–Anaplasma* spp. Conversely, the second article by Morganti et al. (2022), revealed in central Italy a higher prevalence for *L. infantum* (12.2%)*.* A lower prevalence of co-infection with *Anaplasma* spp. (0.6%) and a higher prevalence of co-infection with *E. canis* (1.9%) among seropositive dogs for *L. infantum* was noted*.* Regarding the prevalence of co-infections with *Anaplasma* spp. and *E. canis* in leishmaniotic dogs, the results are consistent with those reported by Vascellari et al. (2016), with no cases identified.

In a study conducted in Central Italy over a 5-year period, in dogs from rural and urban areas, the prevalence of *E. canis* was higher than that of *Anaplasma* spp. (i.e., 7.1% vs 4.68%), and the prevalence of co-infection with *E. canis* and *Anaplasma* spp. was 1.2% (Ebani et al. 2014). These results are in line with the trend of results of Morganti et al. (2022), where a higher prevalence of *L. infantum-E. canis* co-infection is probably due to a high presence of *Rhipicephalus sanguineus* sensu lato ticks, as these vectors adapt to different habitats, and are present in rural and urban environments also completing their entire life cycle indoors, houses, and kennels (Ebani et al. 2014).

Among *L. infantum* seronegative dogs, in Vascellari's study from 2016, the percentage of *Anaplasma* spp. seropositive dogs was higher than the percentage found in Morganti et al. (2022) (i.e., 3.0% and 1.3% respectively). This result is in line with other studies on the occurrence of pathogens transmitted by *Ixodes ricinus* in northern and central Italy, where the prevalence of *A. phagocytophilum* was 7.6% in northern and 10.4% in central Italy (Morganti et al. 2017; Bertola et al. 2021). Furthermore, 2.4% and 2.3% of dogs were seropositive for *E. canis* and no dogs with co-infections by *A. phagocytophilum* and *E. canis* were found.

The findings of the systematic literature review are consistent with those of our study group from the Apulian region. While a higher prevalence of *L. infantum* (58.4%) was observed, the prevalence of co-infections with *A. phagocytophilum* and/or *E. canis* were very low or absent (i.e., no co-infection with *E. canis* alone) (Fig. 2), and there were no significant differences in co-infections with *A. phagocytophilum* and/or *E. canis* in clinically healthy leishmaniotic dogs compared with those serologically negative for *L. infantum*.

The highest prevalence of co-infection observed in this systematic review was reported by Vascellari et al. (2016), where the seropositivity rate for *A. phagocytophilum* was 11.8% in dogs *L. infantum* seropositive. This result differs from the other observed prevalence, which may be influenced by the low cut-off employed in the IFAT test against *L. infantum.* In this case, all dogs co-infected exhibited an antibody titer of 1:40 for *L. infantum.*

The majority of dogs included in both studies resulted from the systematic literature review were CBD and selected based on suitability criteria, which included regular use of anti-feeding/insecticidal products against the main vectors for ectoparasite control. In contrast, none of the dogs in the studied group received treatment with anti-feeding/insecticidal products for ectoparasite control. Thus, the low occurrence of co-infections may be attributed to a reduced circulation of the pathogens *A. phagocytophilum* and *E. canis* in the analyzed areas, as a consequence of a low presence of vectors.

The low prevalence of co-infections in the canine populations analyzed in the two studies resulted from the systematic review and in our study may also be related to climatic conditions, which are known to exert a strong influence on the abundance of ticks and other haematophagous arthropods in the environment and consequently on the circulation of pathogens they transmit. In a 2019 study, Wilke and colleagues (Wilke et al. 2019) posit that global warming will make it possible for ticks to invade new areas that were previously too cold for them to facilitate the establishment. From this perspective, Europe will be particularly affected, with countries such as Germany and Sweden anticipating an increase in the incidence of tick-borne diseases. Furthermore, the authors posit that tick species such as *I. ricinus*, moving northwards, will either disappear or be confined to a few small foci in central and southern Europe due to habitats that are unsuitable for their survival, given the increased aridity. Consequently, there will be no net increase in the prevalence of tick-borne diseases in these areas.

Conversely to the findings of this systematic review, in 2018, Baxarias et al. (2018) stated that dogs diagnosed with clinical leishmaniosis from Catalonia, a Spanish endemic region for CanL and other VBDs, display a higher incidence of co-infections with other VBPs when compared to healthy controls. Out of 61 sick dogs that were previously tested and found to be positive for *L. infantum* through serologic ELISA testing, 34 (56%) and 32 (53%) dogs were respectively found to be positive for *E. canis* and *A. phagocytophilum.* Furthermore, 21 (37%) of these dogs tested positive for both *E. canis* and *A. phagocytophilum* pathogens through IFAT, while 8 (13%) dogs were found to be co-infected with *E. canis* and *Anaplasma* spp. through molecular testing (qPCR). Despite the prevalence rates of co-infections reported, in this study, no statistical association was found between seroreactivity to *E. canis* antigen and sick dogs with leishmaniosis. Thus, the study’s results presented by Baxarias et al. (2018) can be explained for dogs residing in regions with a high incidence of VBDs, particularly those kept outdoors and without regular ectoparasiticide treatment where co-infections occur as a consequence of exposure to arthropod vectors (Roura et al. 2005) and not only due to *L. infantum* inducing immune system suppression or promoting an abnormal response. For example, serological evidence of exposure to multiple vector borne organisms was reported in hunting dogs living outdoors on the island of Mallorca (Solano-Gallego et al. 2006). Nevertheless, it is not possible to make a direct comparison between the results of the Baxarias's et al. study from 2018 and those of this systematic literature review and studied group, as the dog populations analyzed are different (i.e., leishmaniotic dogs displaying clinical signs and clinically healthy *L. infantum* seropositive dogs).

In conclusion, this systematic review presents for the first-time information on the occurrence in Italy of co-infection with *A. phagocytophilum* and *E. canis* in clinically healthy, *L. infantum* seropositive and seronegative dogs, including data from a cohort in southern Italy. The prevalence obtained from the two studies resulted from the systematic literature review and the studied group, showed low and similar percentages of co-infections with *A. phagocytophilum* and/or *E. canis* in both *L. infantum* seronegative and seropositive dogs, suggesting that co-infections may occur independently of *L. infantum* serostatus. These findings emphasize the need for further research to unravel the complex interactions between these pathogens, their vectors, and the host's immune response. A deeper understanding of co-infection dynamics could offer critical insights into the epidemiology and control of vector-borne diseases in endemic areas.

## Data Availability

No datasets were generated or analysed during the current study.
